# Discrete taxa of saprotrophic fungi respire different ages of carbon from Antarctic soils

**DOI:** 10.1038/s41598-018-25877-9

**Published:** 2018-05-18

**Authors:** Kevin K. Newsham, Mark H. Garnett, Clare H. Robinson, Filipa Cox

**Affiliations:** 1 NERC British Antarctic Survey, High Cross, Madingley Road, Cambridge, CB3 0ET UK; 2 NERC Radiocarbon Facility, Scottish Enterprise Technology Park, Rankine Avenue, East Kilbride, Glasgow, G75 0QF UK; 3 School of Earth & Environmental Sciences, The University of Manchester, Manchester, M13 9PL UK

## Abstract

Different organic compounds have distinct residence times in soil and are degraded by specific taxa of saprotrophic fungi. It hence follows that specific fungal taxa should respire carbon of different ages from these compounds to the atmosphere. Here, we test whether this is the case by radiocarbon (^14^C) dating CO_2_ evolved from two gamma radiation-sterilised maritime Antarctic soils inoculated with pure single cultures of four fungi. We show that a member of the Helotiales, which accounted for 41–56% of all fungal sequences in the two soils, respired soil carbon that was aged up to 1,200 years BP and which was 350–400 years older than that respired by the other three taxa. Analyses of the enzyme profile of the Helotialean fungus and the fluxes and δ^13^C values of CO_2_ that it evolved suggested that its release of old carbon from soil was associated with efficient cellulose decomposition. Our findings support suggestions that increases in the ages of carbon respired from warmed soils may be caused by changes to the abundances or activities of discrete taxa of microbes, and indicate that the loss of old carbon from soils is driven by specific fungal taxa.

## Introduction

The mass of organic carbon in soils is estimated to be two to three times that of the carbon which is present in gases (principally CO_2_) in the Earth’s atmosphere^1^. Much of this organic carbon is sequestered in permafrost in cold regions, where sub-zero temperatures and limited water availability restrict decomposition, leading to the accumulation of an estimated 1,330–1,580 pg of the element in soil^2,3^. Restricted decomposition of soil organic matter in cold regions not only results in the accumulation of large stocks of organic carbon, but also results in old, recalcitrant carbon fractions accumulating in soil, with dating of carbon in Arctic soil and in Antarctic deep moss beds yielding estimated ages of up to 2,900–5,350 radiocarbon years before present (BP)^4–6^. It is predicted that significant stocks of soil organic carbon will be respired from active layers of permafrost by soil microbes as the Earth’s climate warms^7^, and so determining the decomposition processes influencing their release to the atmosphere is hence pivotal to our understanding of the carbon cycle and feedbacks on the biosphere.

Central to the decomposition processes that release CO_2_ to the atmosphere from decomposing soil organic matter are heterotrophic microbes such as fungi and bacteria, which secrete extracellular enzymes that cleave bonds in complex organic molecules such as cellulose and lignin^8^. Studies using selective microbial inhibitors consistently indicate that the CO_2_ derived from the fungal catabolism of organic compounds dominates the carbon evolved from soils, with the mean percentage contributions of fungi and bacteria to soil respiration ranging from 60:40–70:30 respectively in arable, grassland and forest soils and litters^9^. It has long been established that specific taxa of fungi are responsible for the decomposition of the different organic carbon fractions present in soils and litters^10,11^. For example, the complex organic polymer lignin is typically decomposed by members of the phylum Basidiomycota, which frequently synthesize lignin modifying enzymes, with cellulose being broken down by a wider range of fungi, typically those belonging to the Basidiomycota and Ascomycota^10–12^. In contrast, fungi such as *Mucor* and *Mortierella* spp., previously placed in the Phycomycetes but now in the Mucoromycotina, usually have more restricted enzyme profiles, and tend to decompose simpler organic compounds such as sugars^12^. More activation energy is required by the enzymes secreted by microbes to degrade organic molecules of increasing complexity^13^, and organic compounds thus have different mean residence times in soils. For example, the half-life in soil of lignin is greater than that of cellulose, which in turn is greater than that of sugars^14,15^. Given that these organic compounds are degraded by discrete taxa of soil fungi^10–12^, it hence follows that there should be differences in the ages of carbon that specific fungal taxa respire to the atmosphere.

Here, as part of wider studies into the microbial ecology of Antarctic soils^16–19^, we couple zeolite molecular sieve technology with ^14^C radiocarbon dating, which in recent years have made it possible to measure the age of carbon in CO_2_ evolved from soil^20^, to test whether or not specific taxa of saprotrophic fungi respire different ages of carbon from sterilised active layer soils sampled from under Antarctic Hairgrass (*Deschampsia antarctica*) at two maritime Antarctic islands. Using molecular techniques, we taxonomically placed the fungi and determined their frequencies in 454 pyrosequencing libraries in order to select taxa that are frequent in the natural environment. By measuring fungal enzyme activities and the δ^13^C content and efflux of CO_2_ respired from soil, we also inferred which soil organic compounds are targeted by each fungal taxon.

## Results

### Taxonomic placement and frequencies of soil fungi

**Figure 1:**
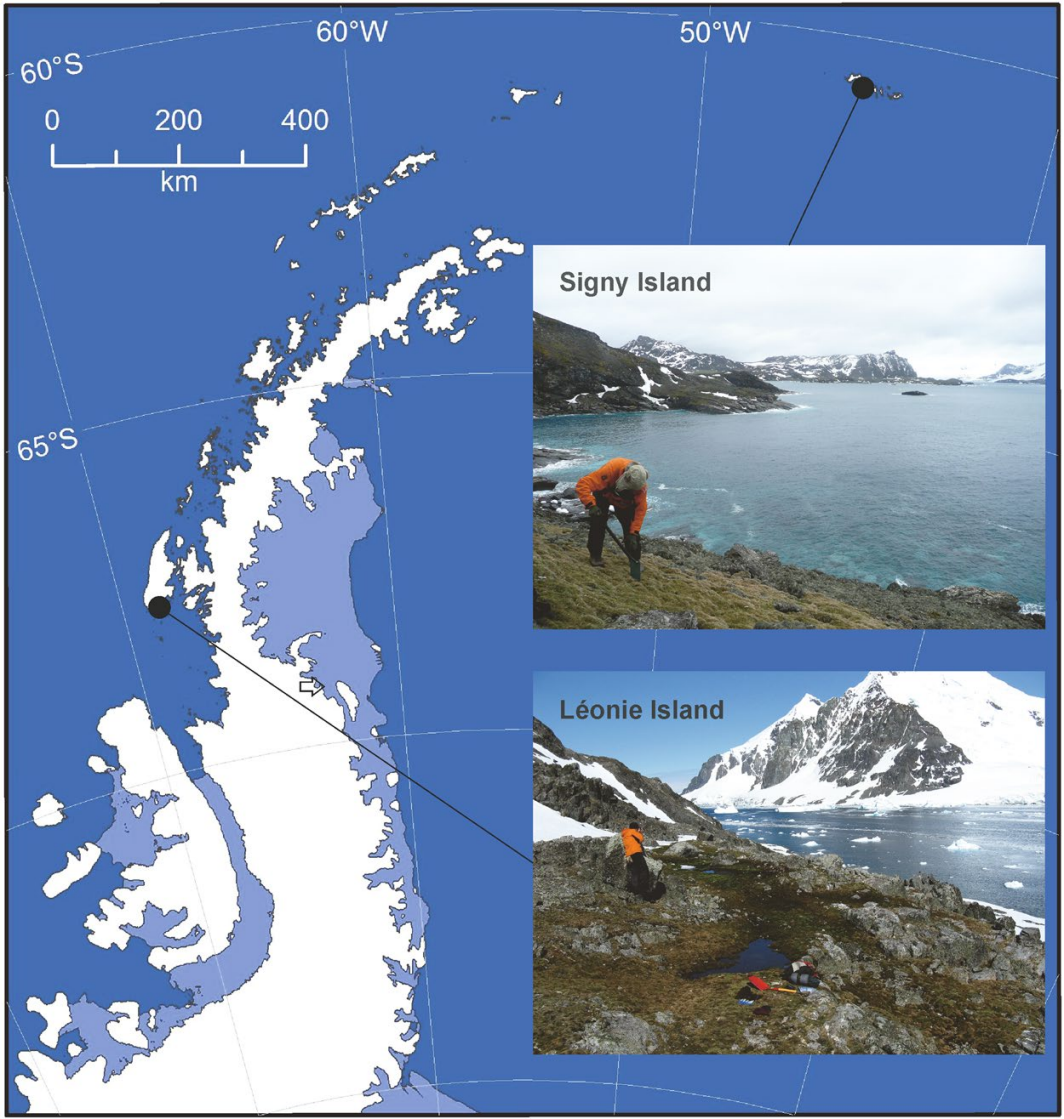
Map of the maritime Antarctic showing the locations of Signy Island and Léonie Island, generated by ArcMap version 10.4 software (http://desktop.arcgis.com/en/arcmap/). Insets show images of the sampling sites.

Blastn searches in the UNITE database indicated that ribosomal DNA internal transcribed spacer (ITS) regions of two fungi isolated from washed roots of *D*. *antarctica* sampled from Signy Island and Léonie Island in the maritime Antarctic (Fig. 1) matched closely with members of the order Helotiales (Table 1). The ITS region of one of these isolates had 99.8% homology with a fungus from soil beneath *Betula nana* in Alaska, but could not be placed below order level and is hereafter referred to as Helotiales sp. 1 (Table 1). The species hypothesis for the second Helotialean isolate, the ITS region of which was identical to that of a fungus from soil beneath *Picea mariana*, also in Alaska, was *Rhizoscyphus* sp. (Table 1). The species hypotheses for the remaining two isolates, the ITS regions of which had 99.8–100% homologies with those of fungi present in North American soil and in the tissues of *Polytrichum strictum* sampled from Antarctica, and both of which were obtained from spread plates, were *Pseudogymnoascus roseus* and *Mortierella turficola*, respectively (Table 1).

**Table 1:** Taxonomic assignments of fungal taxa derived from the UNITE database and their abundances in soil at Signy and Léonie islands, based on the frequencies of DNA reads.

Taxon	Closest UNITE species hypothesis				Frequency of DNA reads in soil (%)	
	NCBI accession no.	SH code	Geographical origin	Substrate	Signy Island	Léonie Island
Helotiales sp. 1	HQ211994	SH013269.07FU	Alaska	Soil	40.62	55.85
*Rhizoscyphus* sp.*	KF617558	SH000129.07FU	Alaska	Soil	0.23	9.84
Pseudogymnoascus roseus**	JX270343	SH006023.07FU	North America	Soil	3.47	0.09
*Mortierella turficola*	HQ335302	SH008900.07FU	Antarctica	Moss tissues	0.90	2.13

*Not inoculated into Signy Island soil; **not inoculated into Léonie Island soil.

Pyrosequencing of fungal DNA indicated that Helotiales sp. 1 accounted for >40% of the fungal DNA reads in soil at both islands (Table 1) and that it was the most frequent fungus in both soils. Based on the frequencies of their DNA in soil, the isolates used in the experiment in total accounted for 45% and 68% of the fungal DNA reads in Signy Island and Léonie Island soils, respectively (Table 1).

### ^14^C analyses of CO_2_

**Figure 2:**
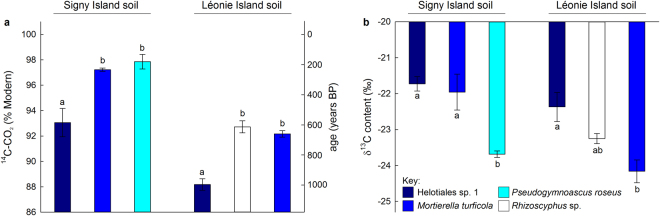
(**a**) ^14^C content (% Modern) and (**b**) δ^13^C (‰) of CO_2_ respired after 50 d from sieved (2 mm) and sterilised soils from Signy Island and Léonie Island inoculated with pure cultures of four saprotrophic fungal taxa. Values are means of 3–4 replicates ± SEM. Distinct letters denote significant (*P* < 0.05) differences between taxa within each soil. Note that neither *y*-axis extends to zero. The right hand *y*-axis in (**a**) shows the age of CO_2_ respired from soil. These data were not subjected to statistical analysis and are shown for comparison only.

The microcosm experiment, in which gamma-irradiated soil from the two islands was inoculated with pure single cultures of the four saprotrophic fungi, indicated that there was a highly significant effect of taxon on % Modern values of carbon respired from soil by the fungi (*F*_3,15_ = 25.14, *P* < 0.001). Helotiales sp. 1 respired CO_2_ from Signy Island soil that was ^14^C-depleted by 4.2–4.8% Modern compared with that respired by *M*. *turficola* and *P*. *roseus* from the same soil (*F*_1,5_ = 38.54, *P* = 0.002 and *F*_1,4_ = 87.87, *P* = 0.001, respectively), approximating to a difference in age of 350–400 years (Fig. 2a). Similarly, CO_2_ respired by Helotiales sp. 1 from Léonie Island soil was ^14^C-depleted by 4.0–4.5% Modern compared with that respired by *Rhizoscyphus* sp. and *M*. *turficola* from the same soil (*F*_1,4_ = 16.16, *P* = 0.016 and *F*_1,5_ = 11.82, *P* = 0.018, respectively), again approximating in each case to an age difference of 350–400 years (Fig. 2a). The single most ^14^C-depleted value recorded was 86.4% Modern, corresponding to an approximate age of 1,171 years BP, measured in CO_2_ evolved by Helotiales sp. 1 inoculated into Léonie Island soil (Supplementary Table S1). After 50 d of incubation in the microcosm experiment, the CO_2_ respired from Léonie Island soil was ^14^C-depleted by 5% Modern compared with that respired from Signy Island soil (mean % Modern values ± SEM: 91.14 ± 0.75 and 96.16 ± 0.71, respectively, *F*_1,15_ = 81.71, *P* < 0.001; Fig. 2a), corresponding to an age difference of approximately 400 years between the carbon respired from the two soils.

### ^13^C contents of CO_2_

After 50 d of incubation, there was a significant main effect of taxon on the δ^13^C contents of CO_2_, with that of the gas respired by Helotiales sp. 1 inoculated into Signy Island soil being ^13^C-enriched by 2.0‰ compared with that respired by *P*. *roseus* from the same soil (mean δ^13^C contents ± SEM: -21.73 ± 0.20‰ and -23.69 ± 0.08‰, respectively; *F*_1,4_ = 81.06, *P* = 0.001; Fig. 2b). Similarly, *M*. *turficola* evolved carbon from this soil with a δ^13^C content of -21.96 ± 0.50‰, which was 1.7‰ more ^13^C-enriched than that in the CO_2_ respired by *P*. *roseus* (*F*_1,5_ = 8.42, *P* = 0.034; Fig. 2b). The CO_2_ respired from Léonie Island soil by Helotiales sp. 1 similarly had a δ^13^C signature that was 1.8‰ more ^13^C-enriched than that of the CO_2_ evolved from the same soil by *M*. *turficola* (mean δ^13^C contents ± SEM: -22.37 ± 0.40‰ and -24.16 ± 0.31‰, respectively; *F*_1,5_ = 12.58, *P* = 0.016; Fig. 2b). There was also a main effect of island on the δ^13^C signatures of CO_2_ evolved by the fungal isolates (*F*_1,15_ = 16.11, *P* = 0.001), with the CO_2_ respired from Signy Island soil being 1.0‰ more ^13^C-enriched than that respired from Léonie Island soil (mean δ^13^C contents ± SEM: -22.40 ± 0.34‰ and -23.35 ± 0.29‰, respectively; Fig. 2b).

### CO_2_ efflux from soil

**Figure 3:**
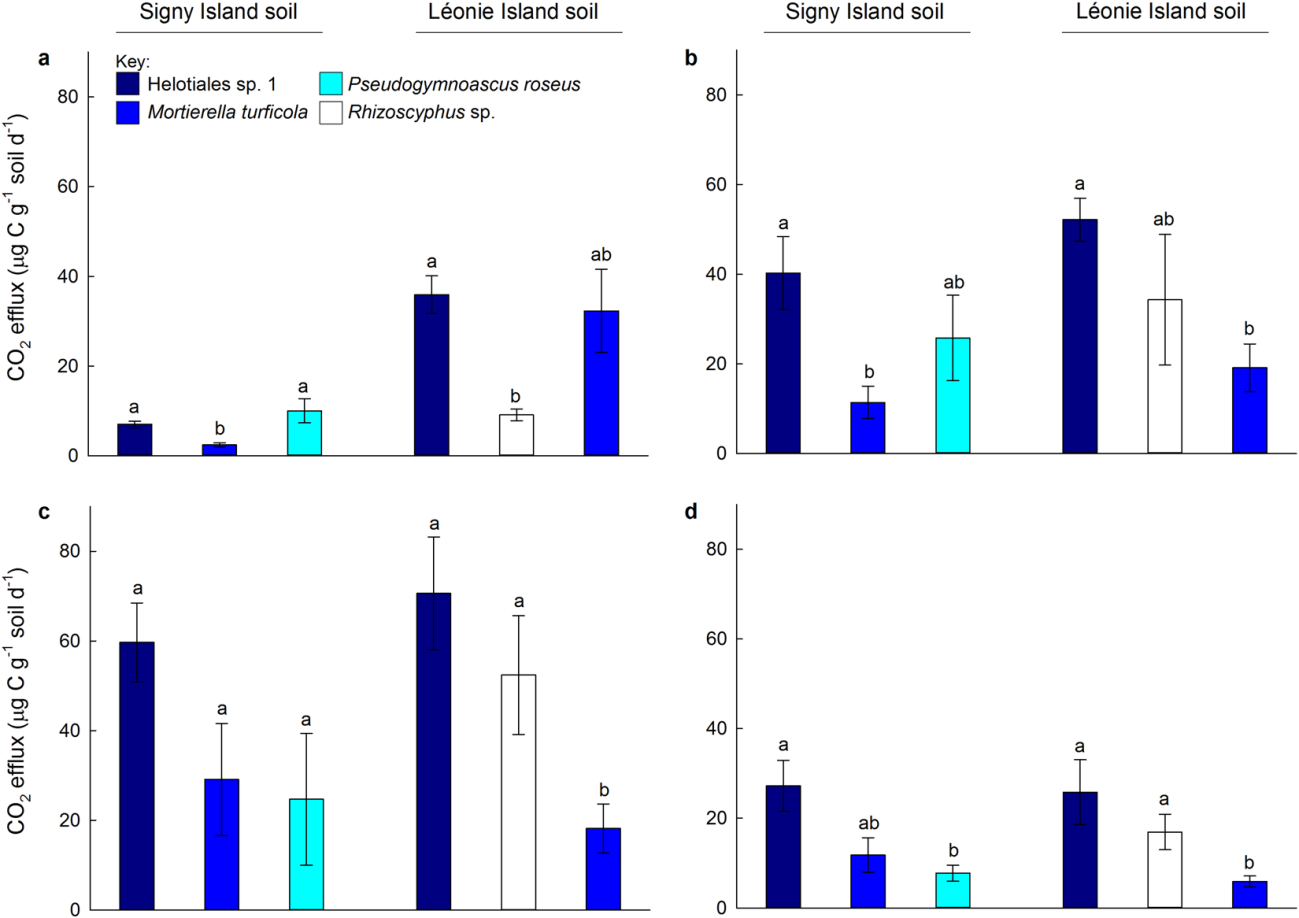
CO_2_ respired after (**a**) 11 d, (**b**) 21 d, (**c**) 27 d and (**d**) 34 d from sieved (2 mm) and sterilised soils from Signy Island and Léonie Island inoculated with pure cultures of four saprotrophic fungal taxa. Values are means of 3–4 replicates ± SEM. Distinct letters denote significant (*P* < 0.05) differences between taxa within each soil.

CO_2_ efflux increased in the microcosm experiment between 11 d and 27 d, with flux rates of the gas declining between 27 d and 34 d (Fig. 3a–d). No significant effects of island were recorded on CO_2_ efflux, except at 11 d, when three fold more CO_2_ was respired by the fungi inoculated into Léonie Island soil than by those inoculated into Signy Island soil (*F*_1,18_ = 14.27, *P* = 0.001; Fig. 3a). Significant effects of taxon on CO_2_ efflux were recorded at all samplings. At 11 d, Helotiales sp. 1 and *Pseudogymnoascus roseus* respired 2–4 times as much CO_2_ from Signy Island soil as did *Mortierella turficola* (*F*_1,4_ = 31.42, *P* = 0.002 and *F*_1,5_ = 10.96, *P* = 0.021, respectively; Fig. 3a). At the same sampling, over three times as much CO_2_ was respired from Léonie Island soil by Helotiales sp. 1 than by *Rhizoscyphus* sp. (*F*_1,4_ = 37.04, *P* = 0.004; Fig. 3a). At 21 d, Helotiales sp. 1 respired two to three times the amount of CO_2_ from both soils than *M*. *turficola* (*F*_1,5_ = 19.75, *P* = 0.007 and *F*_1,5_ = 12.92, *P* = 0.016; Fig. 3b). Similar effects were also observed in Léonie Island soil at 27 d and 34 d, when Helotiales sp. 1 respired approximately three times as much CO_2_ from soil than did *M*. *turficola* (*F*_1,5_ = 18.00, *P* = 0.008 and *F*_1,5_ = 10.24, *P* = 0.024; Fig. 3c,d). At 27 d and 34 d, *Rhizoscyphus* sp. also respired two to three times as much CO_2_ from Léonie Island soil than *M*. *turficola* (*F*_1,5_ = 7.10, *P* = 0.045 and *F*_1,5_ = 9.25, *P* = 0.029; Fig. 3c,d). At 34 d, Helotiales sp. 1 also respired approximately three times as much CO_2_ from Signy Island soil than *P*. *roseus* (*F*_1,5_ = 10.61, *P* = 0.031; Fig. 3d).

### Enzyme assays

Quantitative enzyme assays indicated strong synthesis by Helotiales sp. 1 of the cellulase enzyme β-glucosidase, with significantly more β-glucosidase activity in this fungus than in the other isolates (Table 2). *M*. *turficola* produced less β-glucosidase than the other three fungi (Table 2). *Rhizoscyphus* sp. synthesized more of the chitinase enzyme N-acetyl-β-glucosaminidase than Helotiales sp. 1 and *M*. *turficola*, but there were no differences between any of the four fungi in their abilities to synthesize the peptidase enzyme leucine arylamidase (Table 2).

**Table 2:** Substrate utilisation by fungal isolates, determined using *p*-nitrophenyl assays.

Enzyme	Substrate	*p*-nitrophenol liberated (*µ*M h^−1^)*			
		Helotiales sp. 1	*Rhizoscyphus* sp.	*Mortierella turficola*	*Pseudogymnoascus roseus*
β-glucosidase	4-nitrophenyl-β-D- glucopyranoside	286 ± 12^a^	210 ± 8^b^	33 ± 11^c^	180 ± 7^b^
N-acetyl-β-glucosaminidase	4-nitrophenyl-N-acetyl-β-D-glucosaminidase	1 ± 1^a^	80 ± 6^b^	5 ± 1^a^	45 ± 19^a,b^
Leucine arylamidase	4-nitrophenyl-L-leucyl-2-naphthylamide	0 ± 0^a^	3 ± 2^a^	15 ± 8^a^	21 ± 3^a^

*Values shown are means of 3–6 measurements ± SEM. Values in the same row differing at *P* < 0.05 are indicated by distinctly superscripted letters.

**Figure 4:**
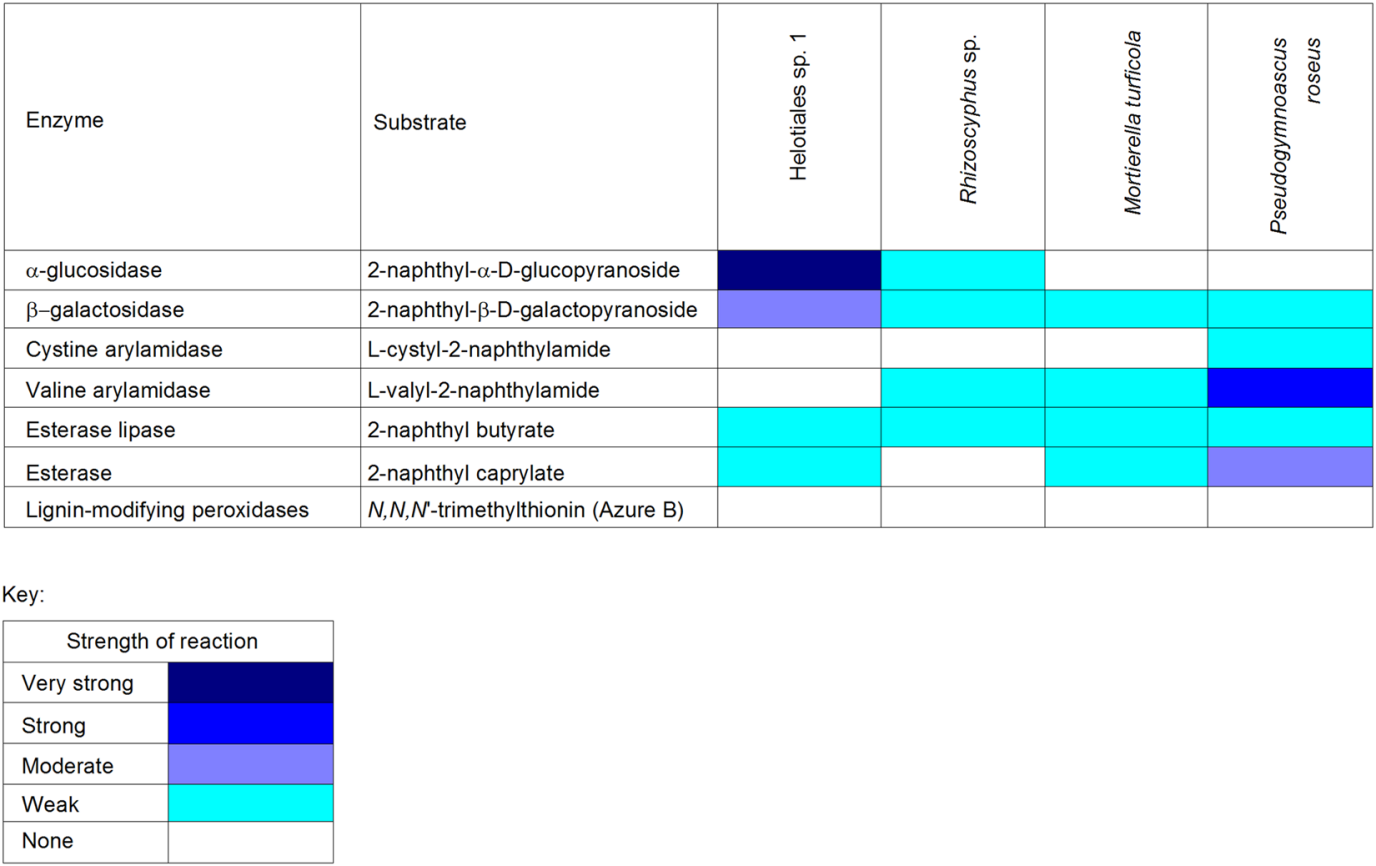
Substrate utilisation by fungal isolates, determined using API ZYM kits and the Azure-B agar medium assay.

Semi-quantitative enzyme assays corroborated the data on cellulase activity derived from the quantitative assays, with analyses of the cellulase enzyme α-glucosidase showing that it was more strongly synthesized by Helotiales sp. 1 than by *Rhizoscyphus* sp. (Fig. 4). These analyses indicated that this enzyme was not produced by *M*. *turficola* and *P*. *roseus* (Fig. 4). The enzyme β-galactosidase was moderately synthesized by Helotiales sp. 1 and only weakly so by the other fungi, whereas the peptidase cystine arylamidase was only weakly produced by *P*. *roseus* (Fig. 4). The other peptidase tested for, valine arylamidase, was strongly produced by *P*. *roseus* and weakly so by *Rhizoscyphus* sp. and *M*. *turficola* (Fig. 4). Esterase lipase was weakly produced by all four fungi, and esterase was only weakly to moderately synthesized by three of the isolates, with *Rhizoscyphus* sp. showing no apparent activity for this enzyme (Fig. 4). Although a temperate region isolate of the wood-rotting Basidiomycete *Trametes versicolor*, which was used as a positive control, degraded the dye *N*,*N*,*N′*-Trimethylthionin, none of the four Antarctic fungi showed any activity of peroxidase-type lignin modifying enzymes (Fig. 4).

## Discussion

The experiments reported here indicated significant differences between saprotrophic soil fungal taxa in the ages of carbon that they respired from two Antarctic soils. A member of the Helotiales was found to evolve CO_2_ that was 350–400 years older than that evolved by the other fungal taxa tested, with the carbon having been fixed from the atmosphere up to 1,200 years BP. Demonstrations of specific taxa of heterotrophic microbes respiring different ages of carbon from soil have not, to the best of our knowledge, hitherto been reported in the literature. Our observations support the view that soil biology has a key role in determining the age of carbon respired from soil^21^, and, just as previous studies have shown that the species composition of primary producer communities can affect the input of carbon into terrestrial ecosystems^13^, they indicate that the composition of heterotrophic soil microbial communities can affect the age of carbon respired from soils to the atmosphere.

We postulate that the respiration of older carbon from soil by Helotiales sp. 1 was owing to its efficient decomposition of cellulose, with the enzyme assays indicating strong synthesis by this fungus of the cellulase enzymes α-glucosidase and β-glucosidase. Given that undecomposed plant tissues were frequent in both soils, it is likely that a substantial proportion of the organic carbon in the soils was present in cellulose, which is known to be frequent in peats formed under Antarctic plants^6^. Consistent with this view, Helotiales sp. 1 typically respired more CO_2_ from soil than *Mortierella turficola*, which only weakly synthesized cellulase enzymes. In accordance with previous studies^22^, differences in the δ^13^C contents of CO_2_ evolved by the fungi also suggested the utilisation of different soil carbon sources. Accurate predictions from such analyses of precisely which soil substrates were utilised by the fungi are fraught with difficulty, owing to the isotopic fractionation of carbon during decomposition^23^. Nevertheless, in agreement with the enzyme assays, the mean δ^13^C contents of CO_2_ respired from soil by Helotiales sp. 1 ranged between −21.7‰ and −22.4‰, which are slightly more ^13^C-enriched than cellulose in herbaceous C3 plant species (−22.5‰ to −24.4‰^15^). Although the CO_2_ respired from Signy Island soil by *M*. *turficola* and Helotiales sp. 1 did not differ in its δ^13^C signature, *M*. *turficola* in Léonie Island soil and *P*. *roseus* in Signy Island soil evolved CO_2_ that was significantly more depleted in ^13^C than that evolved by Helotiales sp. 1, with mean δ^13^C values of −24.2‰ and −23.7‰, respectively. These δ^13^C values are closer to those of other organic components of C3 plant litter in soil, such as lipids, sugars and protein derivatives, which have bulk δ^13^C values of between −24.4‰ and −27.9‰^15^, and which the enzyme analyses here showed were degraded by *M*. *turficola* and *P*. *roseus*. Although we can presently only use measurements of the δ^13^C contents of CO_2_ to infer which soil organic carbon fractions are targeted by distinct fungal taxa, future studies should aim to measure the % Modern values of CO_2_ respired by different fungi decomposing individual components of soil organic matter (i.e., cellulose, lignin, lipids, sugars, proteins and their derivatives). Such measurements would facilitate an accurate view of how different taxa of fungi influence the ages of carbon respired to the atmosphere from soil.

All of the available data in the literature on the ages of carbon evolved from soils are derived from experiments in which CO_2_ has been sampled from bulk soil hosting potentially huge numbers of microbial taxa^21,24–26^. These experiments have usually focused on the effects of warming on the release of old, recalcitrant carbon from soils, and have often led to the conclusion that warming increases the age of carbon evolved from soil^21,25–28^. However, the explanations put forward to account for these increases in the age of carbon released from warmed soil have normally centred on the effects of warming on the fractions of soil carbon that can be assimilated by microbes. While we cannot discount that the release of old carbon from soil may be attributable in part to such factors, the data reported here strongly suggest that it may be owing to increases in the abundances or activities of specific soil microbial taxa under warming treatments. This view is supported by the significant shifts observed in the compositions of root-associated symbiotic fungal communities exposed to warming treatments^29^, the proportional increases in old carbon incorporated into fungal phospholipid fatty acids in warmed soils^27^, and the stimulation by warming of soil microbial genes responsible for the degradation of complex carbon sources in soil, such as cellulose and hemicellulose^28^. Given the abundance of the Helotiales and its parent class the Leotiomycetes in soil, particularly in tundra and boreal forests^30^, and the reported increases in the frequencies of the Helotiales in warmed Arctic soils^31^, this raises the possibility that previously reported increases in the ages of carbon respired from warmed soils^21,25–28^ may have been in part owing to greater abundances or activities of these lineages of fungi.

At odds with the view that only a limited proportion (<20%) of the soil fungal community can be cultured^32^, we were able to isolate fungi accounting for 45–68% of the fungal reads in DNA libraries constructed from both Antarctic soils. This was principally owing to the frequency of Helotiales sp. 1 in the natural environment, which pyrosequencing analyses indicated was the most abundant member of the soil fungal community at both of the Antarctic islands, and which *in vitro* CO_2_ efflux measurements indicated was the most active of the fungi tested in degrading soil carbon compounds. A species of *Rhizoscyphus*, another member of the Helotiales, was also found to be more frequent and active in Léonie Island soil than *M*. *turficola*. This corroborates previous findings that members of the order Helotiales, including *Rhizoscyphus* and *Leptodontidium* spp., are frequent and active members of Antarctic soil and root fungal communities^16^. For example, *Oculimacula yallundae* and species of *Mollisia* and *Tapesia*, all members of the Helotiales inhabiting Antarctic soils, have been shown to be capable of breaking down casein hydrolysate, an organic nitrogen source, leading to the enhanced growth of *Deschampsia antarctica*^17^. Helotialean fungi are also known to be active in fungal decomposer communities at lower latitudes. In experiments similar to those reported here, a member of the Helotiales (a putative *Lemalis* sp.) and *Mucor hiemalis*, which, along with *Mortierella* species, is one of the former Phycomycetes, were inoculated in pure culture onto gamma irradiated petioles of *Pteridium aquilinum*^12^. After six months, the putative *Lemalis* sp. had degraded α-cellulose, hemicelluloses, lignin and soluble carbohydrates, leading to a 7% loss of total carbon, whereas *M*. *hiemalis* had only removed soluble carbohydrates, and consequently did not effect significant loss of carbon from the petioles^12^.

It is appropriate to issue two caveats regarding the data reported here. The first of these is that fungi in the natural environment grow in competition with each other, and not in pure culture, as they did in the microcosm experiment. Competition between fungi for carbon sources may influence soil CO_2_ efflux^33^, decrease carbon use efficiency^34^ and possibly affect the age of carbon respired from soils in an as yet unknown manner. The second caveat is that although we recorded carbon respired from Léonie Island soil aged up to 1,200 years BP, which is older than carbon in Arctic soil at a depth of 10 cm (515 calibrated years BP^35^) or southern Chilean soils at 25 cm depth (177 calibrated years BP^36^), but is in broad agreement with the age of carbon in a shallow (0.2–0.5 m) core through a maritime Antarctic moss bank (1,680 years BP^4^), it remains to be determined if carbon of similar age is respired from maritime Antarctic soils in the natural environment. It is possible that the sieving and gamma irradiation of soil, which were necessary in order to obtain sterile and sufficiently homogenised material for the ^14^C and ^13^C analyses reported here, may have led to structural changes to carbon fractions, possibly increasing the accessibility of previously occluded carbon in organic matter^37^, which is consistent with labile carbon being present in Léonie Island soil for more than a millennium. Given these caveats, although the experiments reported here serve as a valid comparison of the abilities of soil fungal taxa to degrade carbon compounds of different ages in soil, we are cautious about using them to predict the ages of carbon respired by fungi from soils in the natural environment.

Whilst none of the fungal isolates used in the present study were members of the phylum Basidiomycota, and were thus unable to synthesize peroxidase-type lignin modifying enzymes, we postulate that basidiomycete fungi may have important roles in respiring ancient carbon from soils in cold regions. Evidence for this comes from ^14^C analyses of CO_2_ released from Arctic soils^24^, showing the release of older carbon from soil under birch (*Betula pubescens* ssp. *czerepanovii*), the roots of which form ectomycorrhizas with basidiomycetes, compared with the carbon respired from soils under neighbouring tundra heath dominated by *Empetrum hermaphroditum*, an ericoid plant species with roots forming mycorrhizas with ascomycete symbionts^38^. Furthermore, warming has been found to increase the frequencies of ectomycorrhizas formed by the basidiomycete genus *Cortinarius* on the root tips of *Betula nana* in Alaskan soil^29^, potentially leading to the release of ancient carbon^5^. Given the recalcitrance of lignin^14,15^ - derivatives of which are present in both of the soils studied here (C. Horrocks, pers. comm.) - and, despite none of the fungi being able to break down the polymer, the age of carbon respired from both soils approaching 1,200 years BP, it seems plausible that lignin in Léonie Island and Signy Island soils may have been formed millennia ago. We advocate measuring the ages of carbon in CO_2_ evolved by basidiomycetes from Antarctic, Arctic and other cold regions soils, with a view to determining the effects of future climate warming, predicted under moderate greenhouse gas emission scenarios^39,40^, on the release of ancient carbon to the atmosphere.

## Methods

### Soil collection

Active layer soil overlying permafrost (present at *c*. >0.1 m) was collected from depths of 0.02–0.08 m from three pits (0.40 × 0.40 m) dug under turves of the C3 grass *Deschampsia antarctica* in October and November 2011 at Polynesia Point on Signy Island (60.7107° S, 45.5849° W) and on the north-western side of Léonie Island (67.5984° S, 68.3561° W) in the maritime Antarctic (Fig. 1). Soil was not sampled from a depth of 0–0.02 m because of the profusion of live roots at this depth. The majority of the soil was frozen at -20 °C, with sub-samples being frozen at −80 °C, before return to the UK at the same temperatures.

### Gamma irradiation of soils

In order to obtain soil that was sufficiently homogenized for the CO_2_ efflux and ^14^C and ^13^C analyses described below, soil stored at −20 °C was defrosted, passed through a 2 mm sieve and mixed thoroughly. The majority of the soil that was processed in this way passed through the sieve, but some larger fragments of undecomposed plant material were excluded. Sub-samples of soil (250 g fresh weight) were then sterilised with gamma radiation (35 kGy), which, unlike other forms of sterilisation such as autoclaving, is considered to have a minimal effect on soil structure and organic matter^41,42^.

### pH, organic matter and soil moisture concentrations

Nine replicate soil samples from each island that had been frozen at -20 °C were analysed for pH, organic matter and soil moisture concentrations. Soil pH was measured by adding approximately the same volume of deionised water to each soil sample to generate slurries^43^ and recording pH using a glass electrode. Soil moisture was measured by heating *c*. 1 g of fresh soil to constant weight at 105 °C for 17 h, prior to weighing, and organic matter concentrations were measured by heating the dried soil to 550 °C for 4 h, also prior to weighing^44^. These analyses indicated that soil from Léonie Island and Signy Island had mean pH values of 4.5 and 4.3, moisture concentrations of 61.0% and 62.1%, and organic matter concentrations of 80.6% and 74.6%, respectively.

### Isolation and taxonomic placement of saprotrophic soil fungi

Fungi for use in the experiments were isolated onto soil extract medium using spread plates and a modified root washing method^45^. The soil extract was made by adding natural mineral water (1 L) to thawed soil (200 g) from each island and allowing the mixture to settle. The supernatant (815 ml) was filtered and mixed with natural mineral water (185 ml), sucrose (1 g), KH_2_PO_4_ (0.2 g), yeast extract (0.1 g) and Oxoid bacteriological agar no. 1 (15 g) prior to sterilisation by autoclaving. Novobiocin (0.05 g) and Rose Bengal (0.067 g) were also added to each litre of soil extract medium to slow the growth of bacteria and rapidly-spreading fungi. For the spread plates, soil (*c*. 5 mg) was distributed over soil extract medium in 90 mm diameter non-vented Petri dishes under sterile conditions. For the root washing method, roots and decaying organic matter were picked out from the soil, were placed in 30 ml capacity sterile Falcon tubes, and were washed 30 times in sterile water (10 ml) on a vortexer set to maximum speed (50 rev. s^−1^). Between washes, which lasted for 5 min., the water was drained from the material on sterile 1 mm meshes under sterile conditions. After the final wash, the material was blotted dry on sterile filter paper, was cut using a sterile scalpel into 1–2 mm^2^ pieces (organic matter) or 1–2 mm lengths (roots), and was pressed into soil extract medium in 90 mm diameter non-vented Petri dishes. The dishes from both methods were then incubated in the dark at 7 °C for up to 16 weeks. Different morphotypes of fungi were isolated onto, and maintained on, half strength potato dextrose agar (PDA) medium.

A total of 63 morphotypes of fungi were isolated from Léonie Island and Signy Island soils. The fungi were taxonomically placed by sequencing of ITS regions, the universal DNA barcoding marker for fungi^46^. Briefly, DNA was extracted from cultures of each isolate using a commercially available kit (Extract-N-Amp Plant PCR, Sigma Aldrich, St Louis, USA) and was amplified using the ITS1F (5′-CTTGGTCATTTAGAGGAAGTAA-3′^47^)/ITS4 (5′-TCCTCCGCTTATTGATATGC-3′^48^) primer set before sequencing at a commercial facility, using the same primers. Consensus sequences derived from these analyses were taxonomically placed using blastn searches in the UNITE database (https://unite.ut.ee/), with the closest species hypothesis^49^ for each fungus being recorded.

### Pyrosequencing of soil fungal DNA

The fungal isolates used in the microcosm experiments were selected for inclusion based on the number of sequences of each taxon recovered from soil at each island, determined by 454 pyrosequencing of fungal DNA, described in detail elsewhere^19^. Briefly, DNA was extracted from five individual 50 mg sub-samples of soil sampled from Léonie Island and Signy Island that had been frozen at -80 °C after collection using a DNA Elution Accessory kit (MoBio Laboratories, Carlsbad, CA, USA). The DNA was amplified in triplicate PCR reactions using the ITS1F and ITS4 primer set. The ITS4 primer was modified with the 454 A adapter and a 10-bp barcode specific to each sample, allowing identification of different samples once pooled, and the ITS1F primer was modified with the 454 B adaptor. The triplicate PCR products were pooled and subsequently purified using AMPure XP bead purification (Beckman Coulter Inc., Brea, CA, USA) and quantified using Qubit dsDNA HS assays (Life Technologies, Carlsbad, CA, USA) before normalization to consistent concentration. The purified and normalized PCR products were run on a 454 Roche Titanium FLX platform.

The resulting sequences were analysed through the QIIME pipeline^50^. Sequences were filtered to remove reads of low quality or those of <300 bp or >1200 bp, and were split according to barcodes. The remaining sequences were denoised to reduce the influence of characteristic errors associated with 454 pyrosequencing, using the denoiser algorithm available in QIIME^51^, and checked for potential chimeras using UCHIME^52^. Abundant sequences flagged as potential chimeras through either denovo or reference based searches were manually checked. Confirmed chimeric sequences were filtered from the dataset. The ITS2 regions of the remaining high quality non chimeric sequences were extracted using ITSx^53^ to remove flanking conserved regions that can interfere with downstream sequence clustering. The ITS2 sequences were grouped into Operational Taxonomic Units (OTUs) at 97% sequence similarity using USEARCH 6.1^52^, approximating to species-level groupings. OTUs represented by a single sequence were removed from subsequent analyses, since these may often represent erroneous sequences.

### Microcosm experiment

Each 250 g sub-sample of gamma irradiated soil was added to a sterile 1.1 L capacity glass microcosm vented with two 0.2 µm PTFE membrane filters to eliminate contamination by airspora. Each microcosm was inoculated with a single taxon of fungus in pure culture by mixing fresh fungal mycelia (0.3–1.6 g), gently scraped from the surfaces of colonies growing on sterile cellophane discs overlaying half strength PDA medium, into the soil. The three most frequent culturable taxa at each island were inoculated as single pure cultures into the two gamma-irradiated soils. Two of the isolates were inoculated as single cultures into both Léonie Island and Signy Island soils, and the other two isolates were each only inoculated into one soil (Table 1). A total of 24 microcosms were prepared, consisting of three to four replicate microcosms per isolate per soil (Supplementary Table S1) and two control microcosms containing uninoculated gamma-irradiated soil from each island, used as blanks for the CO_2_ efflux analyses (see below). The microcosms were incubated in a laboratory-based system consisting of four 400 W thermocirculators, each attached by tubing to four open-topped water baths and housing four microcosms^54^. In order to approximate temperatures to which soils are exposed during the Antarctic summer^55^, the microcosms were exposed for 50 d to a diurnal cycle in temperature of between 3 °C and 5 °C, with a weekly freeze-thaw event consisting of several hours at -2 °C (Supplementary Fig. S1). The same temperature cycles were applied to soils from both islands because, despite Signy Island and Léonie Island being separated by almost seven degrees of latitude (Fig. 1), long-term climate records indicate that temperatures recorded close to the two islands are similar^56^ owing principally to extensive cloud cover at the more northerly Signy island, and frequent periods of cloudless skies at Léonie Island during the austral summer.

At the end of the experiment, soil (*c*. 5 mg) from each microcosm was placed onto the surface of half strength PDA in 90 mm non-vented Petri dishes under sterile conditions and the dishes incubated at 4 °C for 30 d. All of the fungi that were inoculated into the microcosms were recovered. The soil in the uninoculated control microcosms showed no signs of microbial contamination. The moisture concentrations of the soils from Léonie Island and Signy Island at the end of the experiment were 58.7% and 58.2%, respectively.

### CO_2_ efflux from soil

CO_2_ concentration inside each microcosm, which was sealed with high vacuum grease to ensure that it was gas-tight, was measured at 11, 21, 27 and 34 d after inoculation. Each microcosm was sealed for 24 h before 40 ml of air was extracted from it into a gas-tight syringe, backfilling with the same amount of CO_2_-free air. The gas inside the syringe was injected into an infrared gas analyser in static sampling mode, and the measured CO_2_ concentration recorded. Standards of known CO_2_ concentration and CO_2_-free air were used to verify the IRGA measurements, with CO_2_ concentrations in the uninoculated blanks confirming that the microcosms were gas-tight.

### ^14^C and ^13^C analyses of CO_2_

Samples of respired gas for radiocarbon (^14^C) analysis were collected from the microcosms 50 d after inoculation. The incubation vessels were sealed and atmospheric CO_2_ removed by pumping the headspace gases through a cartridge containing soda lime. The vessels were left until sufficient CO_2_ (>3 ml) had accumulated and were then sampled using molecular sieve traps^20^ by pumping the headspace gases in a closed loop through a molecular sieve trap and back into the vessel. As the headspace gases passed through the molecular sieve, CO_2_ was trapped while others were returned to the incubation vessel. The traps were sealed and transported to the NERC Radiocarbon Facility (East Kilbride, UK), where the CO_2_ was recovered by heating and cryogenic purification^57^ and split into aliquots. The first aliquot was used to determine δ^13^C_VPDB_ by isotope ratio mass spectrometry (Thermo Fisher Delta V, Dreieich, Germany). The second was converted to graphite by Fe-Zn reduction^58^ and analysed for ^14^C by accelerator mass spectrometry (AMS) at the Scottish Universities Environmental Research Centre AMS Facility (East Kilbride, UK). Following convention, ^14^C results were normalised to a δ^13^C of -25‰ (to account for mass dependent fractionation) and expressed as conventional radiocarbon years BP (in which 0 BP = AD 1950) and % Modern (years BP = −8033 × ln [% Modern/100]^59^).

### Enzyme assays

Three methods were used to determine the synthesis by pure cultures of each fungus of enzymes involved in the degradation of selected soil carbon compounds. Quantitative measurements of the activities of three hydrolytic enzymes were made using *p*-nitrophenol assays^60^. Briefly, enzymes were extracted from eight discs (6 mm diam.) of half strength PDA medium, cut from the margins of 14 day old colonies, in 10 mM sodium phosphate buffer (4 ml) adjusted to pH 7.2, which was shaken gently for 100 min. at 4 °C. The activities of cellulase, chitinase and peptidase enzymes were measured using the substrates 4-nitrophenyl-β-D-glucopyranoside (5 mM), 4-nitrophenyl-N-acetyl-β-D-glucosaminide (5 mM) and 4-nitrophenyl-L-leucyl-2-naphthylamide (2.5 mM), respectively. Enzyme extracts (40 μl), substrate solutions (40 μl) and 50 mM sodium acetate buffer adjusted to pH 5.5 (20 μl) were pipetted into wells of a microtitre plate and incubated at 37 °C for 150 min., along with appropriate controls. The reaction was stopped by adding 1 M sodium carbonate (10 μl) to each well. After 3 min., increases in optical density at 405 nm, resulting from the liberation of *p*-nitrophenol by the enzymatic hydrolysis of the substrate, were measured on a plate reader. The amount of *p*-nitrophenol liberated was calculated from a calibration curve of absorbance at 405 nm against known *p*-nitrophenol concentrations.

API ZYM kits (bioMérieux Ltd., Basingstoke, UK) were used to semi-quantitatively assay for six enzymes. The enzymes were extracted from eight discs (6 mm diam.) of half strength PDA medium cut from the margins of 14-day-old colonies. The discs were placed into 0.85% NaCl (2 ml) and shaken at 15 °C for 100 min. Each well of an API ZYM strip was then inoculated with 65 μl of extract from each isolate and colour development was recorded after incubation at 37 °C for 4 h. Finally, peroxidase-type lignin modifying enzymes were qualitatively assayed for by inoculating discs (6 mm diam.) of half strength PDA medium cut from the margins of 14 day old colonies onto Azure-B agar medium and checking for clearance of the dye *N*,*N*,*N′*-Trimethylthionin after 42 d^61^. A culture of *Trametes versicolor*, a wood-rotting Basidiomycete isolated from a temperate habitat, was used as a positive control in this test.

### Statistical analyses

General Linear Models were used to determine the main effects of island and fungal taxon on CO_2_ efflux from soil and the % Modern values and δ^13^C contents of CO_2_, following Anderson-Darling normality tests. Means of these response variables, and of the amount of *p*-nitrophenol liberated in the quantitative enzyme assays, were compared using one-way ANOVA. Analyses were performed in the MINITAB 17 Package (State College, PA, USA).

## Electronic supplementary material

Supplementary Materials
