# Supplementary material for: Discrete taxa of saprotrophic fungi respire different ages of carbon from Antarctic soils
Source: Sci Rep. 2018 May 18;8:7866. doi: 10.1038/s41598-018-25877-9 (PMC5959846; doi:10.1038/s41598-018-25877-9)
Supplement: Supplementary file 1 — Supplementary Materials [file 41598_2018_25877_MOESM1_ESM.pdf]

Supplementary materials

Discrete taxa of saprotrophic fungi respire different ages of carbon from Antarctic soils

Kevin K. Newsham, Mark H. Garnett, Clare H. Robinson & Filipa Cox

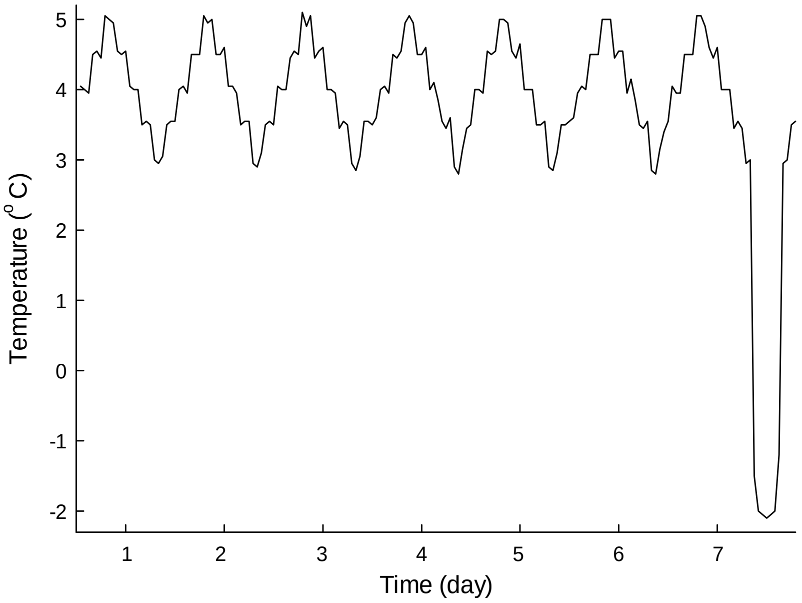

Supplementary Figure S1. Temperatures, recorded hourly with data loggers (Tinytag Plus 2, TGP 4017, Gemini data Loggers, Chichester, UK), to which the soils were exposed.

Supplementary Table S1. ^{14}C enrichment, radiocarbon age and $\delta^{13}C_{VPDB}$ of CO_2 sampled from microcosms.

Island	Fungal taxon	Radiocarbon publication code	^{14}C enrichment (%Modern ± 1σ)	Conventional radiocarbon age (years BP ± 1σ)	$\delta^{13}C_{VPDB}$ (% ± 0.1)
Signy	Helotiales sp. 1	SUERC-63963	93.39 ± 0.43	549 ± 37	-21.9
Signy	Helotiales sp. 1	SUERC-63955	92.17 ± 0.43	655 ± 37	-22.0
Signy	Helotiales sp. 1	SUERC-63966	93.60 ± 0.43	531 ± 37	-21.3
Signy	*M. turficola*	SUERC-63960	96.36 ± 0.44	298 ± 37	-22.8
Signy	*M. turficola*	SUERC-63962	96.46 ± 0.44	289 ± 37	-22.8
Signy	*M. turficola*	SUERC-63944	97.89 ± 0.45	171 ± 37	-21.1
Signy	*M. turficola*	SUERC-63971	98.18 ± 0.45	148 ± 37	-21.1
Signy	*Pseudogymnoascus roseus*	SUERC-63953	97.68 ± 0.45	189 ± 37	-23.8
Signy	*P. roseus*	SUERC-63965	98.35 ± 0.45	134 ± 37	-23.7
Signy	*P. roseus*	SUERC-63954	97.54 ± 0.45	200 ± 37	-23.5
Léonie	Helotiales sp. 1	SUERC-63951	86.43 ± 0.40	1171 ± 38	-22.6
Léonie	Helotiales sp. 1	SUERC-63943	87.82 ± 0.41	1043 ± 37	-23.0
Léonie	Helotiales sp. 1	SUERC-63950	90.27 ± 0.42	822 ± 37	-21.6
Léonie	*Rhizoscyphus* sp.	SUERC-63970	92.83 ± 0.43	597 ± 37	-23.3
Léonie	*Rhizoscyphus* sp.	SUERC-63952	92.46 ± 0.43	629 ± 37	-23.0
Léonie	*Rhizoscyphus* sp.	SUERC-63946	92.86 ± 0.43	595 ± 37	-23.5
Léonie	*Mortierella turficola*	SUERC-63961	91.89 ± 0.42	679 ± 37	-24.1
Léonie	*M. turficola*	SUERC-63964	92.82 ± 0.41	599 ± 35	-24.8
Léonie	*M. turficola*	SUERC-63945	90.67 ± 0.42	786 ± 37	-24.5
Léonie	*M. turficola*	SUERC-63956	93.29 ± 0.43	558 ± 37	-23.3
